# Marginal adaptation, solubility and biocompatibility of TheraCal LC compared with MTA-angelus and biodentine as a furcation perforation repair material

**DOI:** 10.1186/s12903-020-01289-y

**Published:** 2020-10-29

**Authors:** M. A. Alazrag, A. M. Abu-Seida, K. M. El-Batouty, S. H. El Ashry

**Affiliations:** 1 Department of Endodontic, Faculty of Dentistry, Ain Shams University, Cairo, Egypt; 2 Department of Surgery, Anesthesiology and Radiology, Faculty of Veterinary Medicine, Cairo University, Giza - Giza Square, 12211 Egypt

**Keywords:** Biocompatibility, Biodentine, Furcal perforation, Marginal adaptation, MTA, TheraCal LC

## Abstract

**Background:**

This study evaluated the marginal adaptation, solubility and biocompatibility of TheraCal LC compared with mineral trioxide aggregate (MTA-Angelus) and Biodentine when used as a furcation perforation repair material.

**Methods:**

The marginal adaptation was assessed by scanning electronic microscope and presence of any gap between the dentin surface and filling material in each quadrant of the sample was analyzed at 1000 X magnification. The solubility was measured after one week by the ISO standard method. Biocompatibility was evaluated by the inflammatory response and radiography after one month and three months of repair of experimental furcation perforations in dog's teeth.

**Results:**

There were significant differences in the marginal adaptation, solubility and biocompatibility of the tested materials (*P* < 0.05). TheraCal LC showed the highest frequency distribution of gap presence that was followed by the MTA-Angelus then Biodentine. The least soluble material after one week was TheraCal LC that was followed by the MTA-Angelus and Biodentine. After one month and three months, TheraCal LC showed the highest inflammatory response and highest frequency distribution of radiolucency that was followed by the Biodentine then MTA-Angelus.

**Conclusion:**

Unlike Biodentine, TheraCal LC is incapable of alternating the MTA in furcation perforation repair due to its poor biocompatibility and poor marginal adaptation**.**

## Background

Perforations represent pathologic or iatrogenic connections between the root canal space and the external tooth surface. Furcation perforation of the pulpal floor in multi-rooted teeth results in periodontitis and irreversible attachment loss [1].

Several materials have been applied for repairing furcation perforation such as; reinforced zinc oxide eugenol, amalgam, super EBA, calcium hydroxide, composite resins, glass ionomer, MTA, bioaggregate, biodentine, platelet rich plasma (PRP), platelet rich fibrin (PRF) and others, but none of them has the characteristics of an ideal repair material [1–3]. An ideal perforation repair material should be inducing osteogenesis, cementogenesis and good sealing, nontoxic, noncarcinogenic, biocompatible, dimensionally stable and insoluble in the body's fluids [2, 3].

The MTA is one of the most commonly used root repair materials due to its good biocompatibility, marginal adaptation, bacterial leakage, and low cytotoxicity, but it has some drawbacks such as long setting time (3 h), difficult applicability and expensiveness [1–3]. The MTA-Angelus (Angelus, Londrina, PR, Brazil) is another formulation of ProRoot MTA that has short sitting time (10 min) [4].

Biodentine (Septodont, Saint-Maurdes-Fosses, France) is an alternative to MTA due to its similar properties when compared with the MTA, but with better handling properties and faster setting time [3]. A modified powder constituents, addition of setting accelerators and softeners, and a new predosed capsule formulation are responsible for the improvement of the physical properties of biodentine [5].

In recent years, innovations are still carried out to introduce recent endodontic repair materials that overcome the shortcomings of the available materials. TheraCal LC (Bisco Inc, Schaumburg, IL, USA) is a new light-curing Resin Modified Calcium Silicates (RMCS) material that has been recorded to enhance apatite formation and secondary dentin. Due to its high physical properties and low solubility, TheraCal is applied mainly as a barrier and for protection of dental pulp complex [6]. Therefore, evaluation of the physical and biological properties of this newly introduced resin modified calcium silicate-based material (TheraCal LC) is thought to be valuable.

The marginal adaptation, solubility and biocompatibility are the main tested properties of the furcation perforation repair materials [7–9]. The hypothesis of this study was that TheraCal LC will alternate the MTA and Biodentine as a furcation perforation repair material. Therefore, this study compared the repair potential of TheraCal LC Resin Modified Calcium Silicate material against the MTA-Angelus and Biodentine as a furcation perforation repair material, in terms of marginal adaptation, solubility and biocompatibility.

## Methods

### Materials

#### TheraCal LC (Bisco Inc, Schaumburg, IL, USA)

Light-curing, resin-modified calcium silicate in single paste [Composition: Calcium silicates (Portland cement type III), Bis-GMA (Bisphenol A diglycidyl methacrylate), PEGDMA (Polyethylene glycol dimethacrylate) and Barium zirconate].

#### Mineral trioxide aggregate (Angelus, Londrina, PR, Brazil)

[Composition: Powder: Tricalcium silicate, Dicalcium silicate, Tricalcium aluminate, Tetracalcium aluminoferrite and Bismuth oxide. Liquid: Distilled water].

#### Biodentine (Septodont, Saint-Maurdes- Fosses, France)

[Composition: Powder: Tricalcium silicate, Dicalcium silicate, Calcium carbonate, Iron oxide and Zirconium oxide. Liquid: Water, Calcium chloride and Modified polycarboxylate].

## Evaluation of marginal adaptation

### Sample selection and preparation

Three freshly human extracted single rooted teeth were obtained from patients who received orthodontic treatment at the outpatient clinic, Faculty of Dentistry, Ain Shams University, Egypt. The institutional ethical committee approved the work on the extracted teeth without consents (Protocol No. 349-Endo). The sample size was estimated according to 80% power analysis at 95% confidence interval based on pilot study using G power version 3.1.9.7.

The teeth were cleaned by removing the hard deposits using a curette and the soft tissues by soaking in 5.25% Sodium hypochlorite (NaOCl) solution for 10 min. Sample preparation was performed by removing the crown part and apical segment of each tooth to leave the coronal and middle third measuring 10 mm in length using a high speed hand piece under water spray. The roots were placed in acrylic blocks and three horizontal sections. (2 ± 0.1 mm thick) were made from root segments of each tooth using a diamond disc under continuous water irrigation and the final thickness of each slice was measured with a digital caliper having an accuracy of 0.001 mm (CenTech 4, Harbor Freight Tools, Calabasas, CA, USA). Low speed round bur size 1.2 mm diameter (ISO size 012, Komet Dental, Gebr, Brasseler GmbH &Co. KG, Germany) was used to drill three perforations like cavities parallel to the root canal in each root slice. A minimum distance of 1 mm was maintained between the cavities, the external cementum and the root canal wall. Afterwards, all root slices were immersed in a 2.5% NaOCl solution for 15 min and then immersed in distilled water. The smear layer was removed using 17% Ethylenediaminetetraacetic acid (EDTA) solution for 3 min, distilled water for 1 min, 2.5% NaOCl for 1 min and a final flush with distilled water for 1 min [10]. A 3 mL plastic syringe with a 21G needle was used to apply NaOCl and EDTA solution to the prepared cavities.

The cavities were then dried with absorbent paper and the three cavities of each root slice were randomly filled with one of the selected experimental materials. The cavities filled with TheraCal LC were light cured (Woodpecker Light cure LED Mini-S, Woodpecker medical instrument, Changhai, China) for 20 s while the Biodentine and MTA-Angelus were mixed according to the manufacturer, s instructions as described before and delivered to the cavities with the special MTA carrier. Nine root slices with twenty seven cavities were produced then divided into 3 groups (9 cavities each); group I: filled with MTA-Angelus, group II: filled with Biodentine and group III: filled with TheraCal LC. Each slice was marked with an indelible marker. The filled root slices were incubated in contact with a piece of gauze moistened with saline solution at 37 °C for 24 h for a complete setting of the materials.

### Samples evaluation

All samples were examined by a scanning electron microscope (SEM Model Quanta 250 FEG (Field Emission Gun) attached with EDX Unit, NTS Gmbh, Germany) at low vacuum. The perimeter of each cavity was divided into 4 quarters and the presence of any gap between the dentin surface and filling material in each quadrant was analyzed at X1000 magnification in scanning electron microscope. The marginal adaptation was classified according to Aggarwal et al. [11] into; continuous non gapped margin (continuous interface between the filling material and dentin with less than 1um gap) and gapped margin (interface between the filling material and dentin with gaps more than 1um wide). The SEM photos were transferred to a computer and the gap was measured using Image J software (Image J v1.44; US National Institutes of Health, Bethesda, MD, USA).

## Solubility evaluation

### Classification and preparation of samples

Split Teflon ring Molds (2 mm thick and 8 mm internal diameter) were used to prepare 30 identical specimens. These specimens were divided into three groups (10 specimens each) according to the material used; group I: MTA-Angelus, group II: Biodentine and group III: TheraCal LC. Each group was subjected to solubility and disintegration testing according to the International Standards Organization (ISO) 6876 method [12] and with American Dental Association (ADA) specification No.30 [13].

### Solubility test

The Split Teflon ring Molds were placed on a glass plate covered with cellophane paper while the waxed thread was placed inside the mold that was filled to slight excess with tested materials. After filling the mold, another glass plate covered with cellophane paper was placed on the top of mold exerting light pressure to remove any excess material. The molds were stored in an incubator (Heraeus incubator, West Germany) at 37 °C and 95% humidity then the materials were left to set. The specimens were removed from the molds and the net weight of each specimen was recorded (W0) by high precision electrical weighting balance (Precision electrical weighting balance, Percisa 120A,West Germany) adjusted to give 0.0001-g accuracy.

Each specimen was suspended vertically by the waxed thread in a clean glass beaker filled with distilled water at 37 °C in a special incubator for 1 week. At the end of the period, the specimens were removed from their glass beakers and rinsed with a little distilled water and the surplus water was removed from the specimens by gentle blotting with paper tissues. The specimens were stored in desiccators containing thoroughly dry anhydrous calcium sulfate (CaSO4) for 24 h and reweighed to nearest 0.001gram (W1). Solubility percent for each sample was calculated as follows: % Wight loss = W0—W1/W0 × 100.

## Biocompatibility evaluation

### Animal model

The present study was approved by the Institutional Animal Care and Use Committee at Faculty of Dentistry, Ain Shams University, Egypt (Protocol No. 349-Endo). The authors followed up all institutional regulations and The Animal Research: Reporting in Vivo Experiments guidelines (ARRIVE).

Eight adults clinically free mongrel male dogs were obtained commercially from Al-Fahad Trading Company of Animals (Abu-Rawash, Giza, Egypt). The weight of these dogs was15 to 20 kg and the age was 2 to 3 years. The animals were quarantined in separate cages at Department of Surgery, Anesthesiology and Radiology, Faculty of Veterinary Medicine, Cairo University, Egypt. The dogs were fed, examined and kept under observation of an expert veterinarian for two weeks before they were used as experimental animals in this study.

### Classification of samples

In each dog 10 bi-rooted premolars were used. The animals were randomly divided according to the evaluation period into 2 main groups (4 dogs each); group I: after 1 month and group II: after 3 months. Each group was randomly subdivided into 4 subgroups (10 teeth each) according to the material used for perforation repair as follows; subgroup (A): MTA-Angelus, subgroup (B): Biodentine, subgroup (C): TheraCal LC and subgroup (D): Positive control (perforation without sealing). All subgroups were represented in each dog.

### Surgical procedure

Each dog was anesthetized with general anesthesia after fasting for 12 h using Atropine sulphate (Atropine sulphate®, ADWLA Co., Egypt) at a dose of 0.05 mg/kg injected subcutaneous, Xylazine HCl (Xylaject®, ADWIA Co., Egypt) at a dose of 1 mg/kg injected intramuscular and Ketamine HCl (Keiran®, EIMC Pharmaceuticals Co., Egypt) injected intravenous through a cannula in the cephalic vein at a dose of 5 mg/kg. The anesthesia was then maintained by using a 2.5% solution of Thiopental sodium (Thiopental sodium®, EIPICO, Egypt) at a dose of 25 mg/kg injected intravenous (dose to effect). Preoperative periapical radiograph was taken for each tooth to confirm the absence of periodontal defect and bone loss.

Access cavities were made through the occlusal surface of the bi-rooted premolars using a tapered diamond stone with a conventional speed hand piece mounted on the electric motor. The chamber was irrigated with NaOCl 2.5% and the pulp tissue was removed using a suitable excavator. Size 15 K file was carried into the canals till the apex and the working lengths were confirmed by apex locator. The canals were cleaned and shaped up to the working length using stainless steel hand K files size 35 and 40. The canals were irrigated with NaOCl 2.5% and saline solutions, then dried with paper points. The canals were obturated with gutta-percha cones and resin sealer using the lateral compaction technique.

A perforation was made in the center of the floor of pulp chamber using a sterile, low speed round bur 1.2 mm diameter (Komet Dental, Gebr, Brasseler GmbH &Co. KG, Germany) (ISO size 012) [14] to penetrate the furcation area into the periodontal tissue. The penetration depth was estimated 2 mm into the alveolar bone using a rubber stopper as a marker on the shank of the bur. The observed bleeding was controlled with paper points and the perforations sites were irrigated with saline solution.

The perforations were sealed immediately by one of the three tested materials according to the subgroup. The perforation area was left open without repair in the positive control subgroup. The materials were prepared and mixed according to manufacturer’s instruction as follows:

### MTA-Angelus

One part of water was added to 3 parts of cement, then gradually incorporated the liquid into the cement using a plastic mixing stick for about 1 min. The paste was carried out into the perforation by the special MTA carrier and compacted with a suitable size plunger.

### Biodentine

The capsule was gently tapped on a hard surface to loosen the powder, then opened and placed on the white capsule holder. Then 5 drops of the liquid were poured into the capsule. The capsule was closed and placed on a mixing device (amalgamator) at a speed of 4000 rotations/min for 30 s. Then the material was collected and carried out to the perforation site by the special MTA carrier and compacted with a suitable size plugger.

### TheraCal LC

Ready to use paste in a single syringe (1 g) was injected directly into the perforation site and then cured for 20 s with light cure (MINIS curing light/Woodpecker medical instrument).

The coronal access cavities of all teeth were filled with chemical cured glass ionomer cement as a permanent restoration (Riva Light Cure LC/Southern Dental Industries SDI).

For pain and infection control, all dogs were given intra-muscular injections of Cefotaxime sodium at a dose of 10 mg/kg (Cefotax 250 mg vial®, T3A Co., Egypt) and Diclofenac sodium at a dose of 1.1 mg/ kg (Voltaren 75 amp®, Novartis Co., Egypt) once daily for five postoperative days [15]

### Radiographic evaluation

Two periapical radiographs were taken for each tooth using conventional size 2 dental films (Kodak Ultra-Speed Dental Film®). Postoperative periapical radiograph was taken for each tooth immediately after perforation repair and follow-up periapical radiograph was taken for each tooth after the evaluation period of each group. The radiographs were processed using an automatic film processor (Durr, Periomat plus) and transferred to a PC computer with transparency scanner (HP Scanjet G4050 Hewlett-Packard Development Company, L.P.) then evaluated. According to a previous study by Vanni et al. [16], the evaluation was performed by an experienced operator unaware of different experimental groups with respect to the presence or absence of radiolucency in the furcation region.

### Histopathological evaluation

According to the group, all dogs were euthanized by an anesthetic overdose using 20 mL of 5% Thiopental sodium injected rapidly through the cephalic vein. Both jaws were dissected and sectioned into halves at the midline using a saw. Block sections including the experimental teeth with surrounding bone were obtained. The blocks were fixed in 10% buffered formalin solution with a ratio of 1:50. After two weeks of fixation, the blocks were decalcified by 17% EDTA solution for 120 days. After decalcification, the specimens were prepared as usual and stained with hematoxylin and eosin. The stained sections were blindly examined by a pathologist using a light microscope (OLYMPUS BX60 Microscope, Olympus Inc, Japan) and photographs were taken using a digital camera fitted to the light microscope (Canon EOS 750 D, Canon Inc, Japan). The inflammatory cell count was assessed. For each slide, three microscopic fields were captured at magnification 400X. The inflammation was categorized by counting visible inflammatory cells on each field, according to a previous study [17] as follows:

- Score 0: represented non or few inflammatory cells
- Score 1: represented less than 25 cells
- Score 2: represented 25–125 cells
- Score 3: represented more than 125 cells

### Statistical analysis

Quantitative data were presented as mean, standard deviation (SD) and range (minimum – maximum) for numerical values. Data were explored for normality by checking the data distribution and using Kolmogorov–Smirnov and Shapiro–Wilk tests. ANOVA was used and followed by Tukey’s post-hoc test for pairwise comparisons when ANOVA test was significant. Chi square test was used for categorical data. The significance level was set at *P* = 0.05 and 95% Confidence interval. Statistical analysis was performed using Graph Pad Instat (Graph Pad, Inc.) software for windows.

## Results

### Marginal adaptation of the tested materials (Fig. 1)

**Fig. 1:**
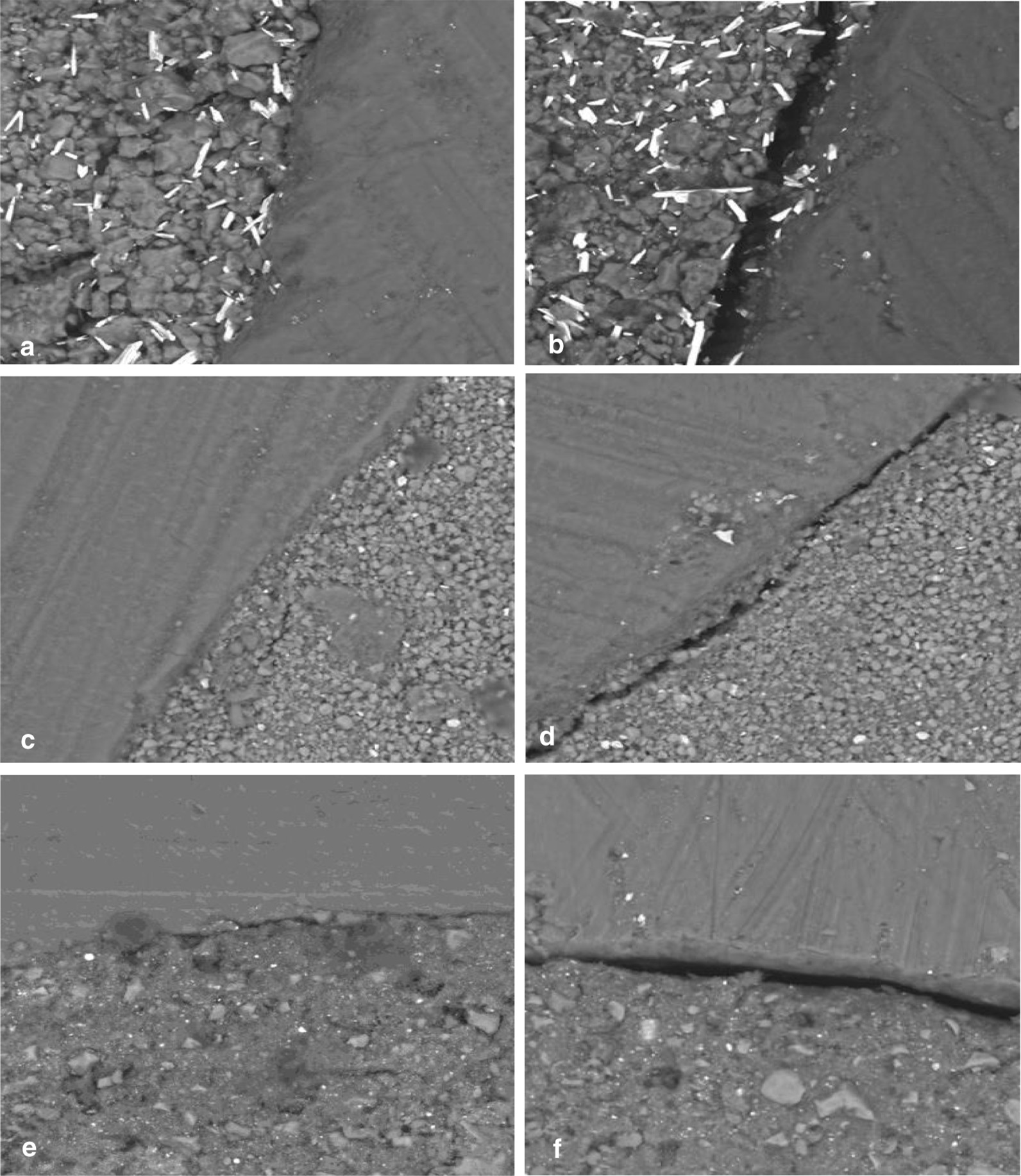
SEM photomicrographs (magnification X1000) of cavities filled with MTA-angelus (**a**, **b**), Biodentine (**c**, **d**) and TheraCal LC (**e**, **f**) showing no gap evident between the materials and dentin (**a**, **c**, **e**) and a gap evident between the materials and dentin (**b**, **d**, **f**)

The highest frequency distribution of gap presence was recorded for the TheraCal LC (gapped = 61% and non-gapped = 39%) that was followed by the MTA-Angelus (gapped = 50% and non-gapped = 50%). While Biodentine exhibited the lowest frequency distribution of gap presence (gapped = 25% and non-gapped = 75%). The difference between the tested materials was significant as indicated by Chi square test (*P* < 0.05).

### Solubility of the tested materials

The highest mean solubility percent was recorded for the Biodentine (3.361178 ± 0.2621%, minimum value = 2.1% and maximum value = 4.3%), that was followed by the MTA-Angelus (mean value = 1.742891 ± 0.396973%, minimum value = 0.51% and maximum value = 3%). While the lowest mean solubility percent was recorded for the TheraCal LC (mean = 1.566595 ± 0.33298%, minimum value = 0.25% and maximum value = 2.3%).

The difference between the tested materials was significant as indicated by one way ANOVA test (F = 60.94, *P* < 0.05). Pairwise Tukey’s post-hoc tests showed non-significant (*P* > 0.05) differences between the MTA-Angelus and TheraCal LC.

### Biocompatibility of the tested materials

#### Presence or absence of radiolucency

**Fig. 2:**
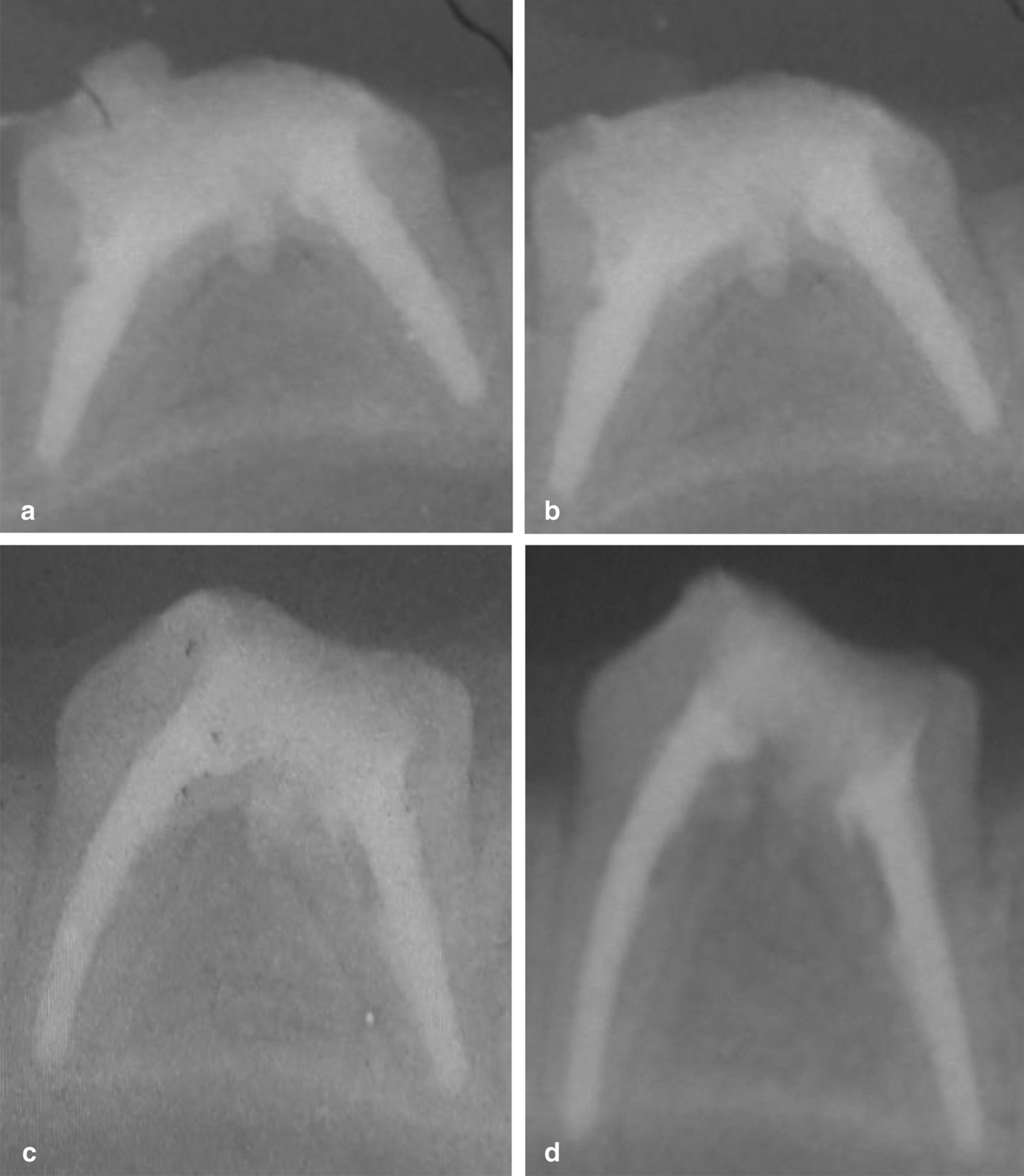
**a** A representative periapical radiograph of the MTA subgroup immediately post perforation repair. **b** Periapical radiograph of the MTA subgroup showing absence of furcal radiolucency and bony defect after 3 months of evaluation. **c** A representative periapical radiograph of the Biodentine subgroup immediately post perforation repair. **d** A periapical radiograph of the Biodentine subgroup showing absence of furcal radiolucency and bony defect after 3 months of evaluation

**Fig. 3:**
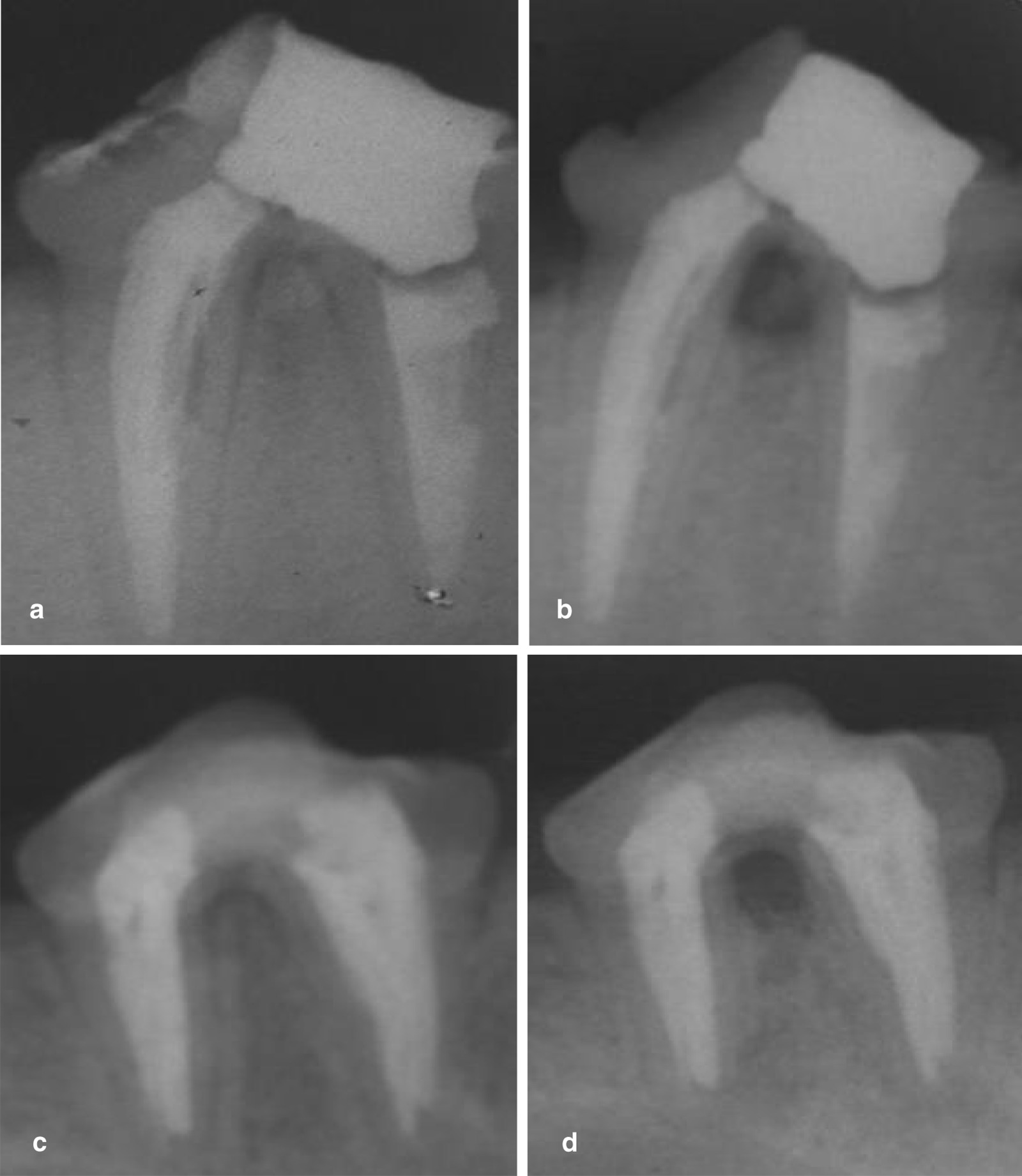
**a** A representative periapical radiograph of the TheraCal LC subgroup immediately post perforation repair. **b** A periapical radiograph of the TheraCal LC subgroup showing presence of furcal radiolucency and bony defect after 3 months of evaluation. **c** A representative periapical radiograph of the positive control subgroup immediately post perforation. **d** A periapical radiograph of the positive control subgroup showing presence of furcal radiolucency and bony defect after one month of evaluation

The frequencies of radiolucency percent for all subgroups after one month and three months are summarized in Table 1. The highest frequency distribution of radiolucency presence was recorded in the control subgroup that was followed by the TheraCal LC subgroup then the Biodentine subgroup (Figs. 2 and 3). While the MTA-Angelus exhibited the lowest frequency distribution of radiolucency presence. The difference in the frequency distribution of radiolucency between subgroups was significant as indicated by the Chi square test (*P* < 0.05). The difference between the MTA-Angelus and the Biodentine subgroups was non-significant at one month and three months and the difference between the TheraCal LC and control subgroups was non-significant at three months (*P* > 0.05).

**Table 1:** Frequency distribution of radiolucency (%) for all subgroups after one month and three months groups

Subgroups	1 month group		3 months group	
	Presence	Absence	Presence	Absence
MTA	10	90	20%	80
Biodentine	20	80	30%	70
TheraCal LC	40	60	90%	10
Control	100	0	100	0
Chi value	199		208	
*P* value	< 0.0001*		< 0.0001*	

*P < 0.05

The difference in the frequency distribution of radiolucency between one month and three months groups for all subgroups was non-significant as indicated by the Chi square test (*P* > 0.05), except for the TheraCal LC subgroup where the difference between one month and three months groups was significant (*P* < 0.05).

#### Inflammatory cell count

**Fig. 4:**
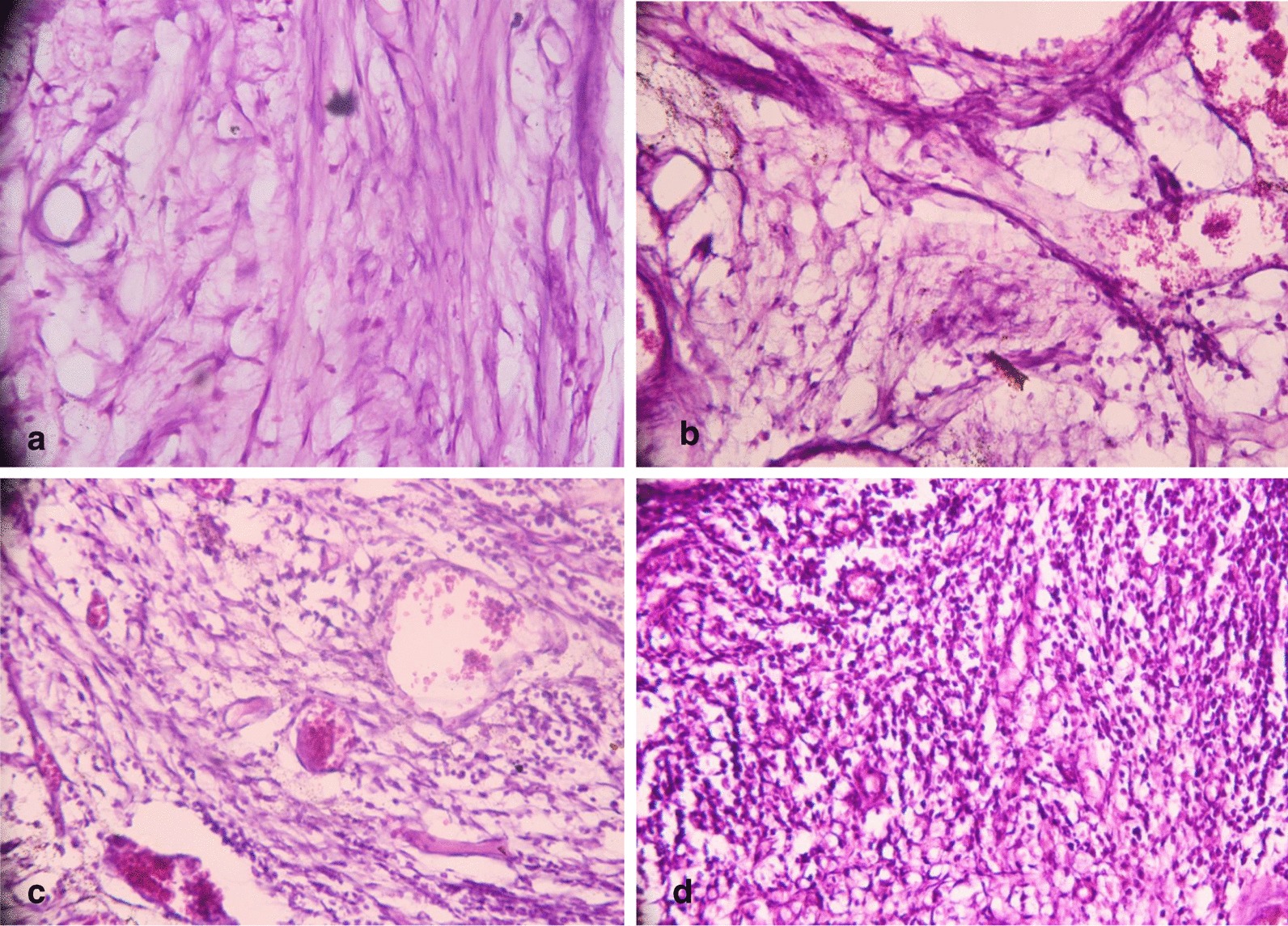
**a** A representative photomicrograph of the MTA-Angelus subgroup showing a score 1 inflammatory cell count after 1 month of evaluation (H&E, × 400).** b** A representative photomicrograph of the Biodentine subgroup showing a score 2 inflammatory cell count after 1 month of evaluation (H&E, × 400).** c** A representative photomicrograph of the TheraCal LC subgroup showing a score 3 inflammatory cell count after three months of evaluation (H&E, × 400). **d** A representative photomicrograph of the positive control subgroup showing a score 3 inflammatory cell count after three months of evaluation (H&E, × 400)

After one and three months, the frequency distributions of inflammatory scores % for all subgroups are summarized in Table 2. The control subgroup exhibited the highest percentage of inflammatory scores that was followed by the TheraCal CL subgroup then the Biodentine subgroup (Fig. 4). The MTA-Angelus subgroup exhibited the lowest percentage of inflammatory scores. The difference in the frequency distribution of inflammatory scores was significant between the subgroups at one month and three months as indicated by the Chi square test (*P* < 0.05).

**Table 2:** Frequency distribution of inflammatory scores percentage for all subgroups after one month and three months groups

Subgroups	Score 0		Score 1		Score 2		Score 3	
	1 month	3 months	1 month	3 months	1 month	3 months	1 month	3 months
MTA	0	0	70%	80	30%	20%	0	0
Biodentine	0	0	60%	70	40%	30%	0	0
TheraCal LC	0	0	50%	10	50%	50%	0	40%
Control	0	0	0	0	60%	60%	40%	40%
Chi value	195.6	230	195.6	230	195.6	230	195.6	230
*P* value	< 0.0001*	< 0.0001*	< 0.0001*	< 0.0001*	< 0.0001*	< 0.0001*	< 0.0001*	< 0.0001*

*P < 0.05

Table 3 shows the effect of time on the inflammatory cell count for different subgroups. The difference in the frequency distribution of inflammatory scores between one month and three months groups for all subgroups was non-significant as indicated by the Chi square test (*P* > 0.05), except for TheraCal LC subgroup where the difference between one month and three months groups was significant (*P* < 0.05).

**Table 3:** Comparison of frequency distribution of inflammatory scores percentage for each subgroup between one month and three months groups

Scores	MTA		Biodentine		TheraCal LC		Control	
	1 month	3 months	1 month	3 months	1 month	3 months	1 month	3 months
Score 0	0	0	0	0	0	0	0	0
Score 1	70%	80%	60%	70%	50%	10%	0	0
Score 2	30%	20%	40%	30%	50%	50%	60%	60%
Score 3	0	0	0	0	0	40%	40%	40%
Chi value	2.16		1.8		66		0	
*P* value	0.1416 ns		0.186 ns		0.0001*		1 ns	

## Discussion

Furcation perforation has usually occurred at the furcal site of the posterior teeth, leading to a bad effect on the prognosis of the affected teeth [18]. In order to decrease the inflammation and enhance the periodontal ligament (PDL) attachment, an ideal furcal perforation repair material should be used. Calcium silicate cements are the materials of choice for treatment of the furcation perforation due to their good biocompatibility and ability to induce calcium-phosphate precipitation at the interface to the periodontal tissue with high quality of the material-dentin interface [19]. Therefore, the present study compared the TheraCal LC with the MTA-Angelus and Biodentine, in terms of marginal adaptation, solubility and biocompatibility. The results of this study recorded significant differences between the tested materials regarding the evaluated properties with superiority of the MTA-Angelus that was followed by Biodentine then TheraCal LC. Therefore, the hypothesis of this study is rejected.

The three-dimensional hermetic seal is one of the most important requirements during furcation perforation repair. This seal is a complex outcome of marginal adaptation, adhesion, solubility, and volume changes of the cement used. Therefore, the gap size between the dentin and repair material and the fluid leakage constitute the quantitative manifestation of the material sealing ability [20]. The evaluation of marginal adaptation of furcation perforation repair materials by SEM can give information about their sealing ability [21]. The results of the present study showed that TheraCal LC exhibited a lower sealing ability than that of the Biodentine and MTA-Angelus and Biodentine exhibited a better sealing ability than the MTA-Angelus. These results agree with that of a previous study [22]. The good adaptation property of Biodentine may be attributed to the small size of Biodentine particles which may enhance the adaptation at the cavity surface and filling interface. On the other hand, the marginal adaptability and microlakage of the MTA were better than that of the Biodentine [20]. The difference between our results and other controversial results may be related to the method of sample preparation and method of evaluation.

As regards the TheraCal LC, it exhibited the highest frequency distribution of gap compared to MTA-Angelus and Biodentine. This poor adaptability might be due to the presence of resin matrix in the TheraCal LC that may maximize its polymerization shrinkage and consequently reflects on the material adaptation. In contrast, our result is not in agreement with the study done by Makkar et al*.* [22] who compared the sealing ability of TheraCal LC, Biodentine and MTA by using the dye penetration method and Confocal Laser Scanning Microscope for evaluation. They found that TheraCal LC exhibits less microleakage than other materials. This difference in the results could be related to the difference of sample preparation and the evaluation method. Therefore, it is worth to mention that in comparing the results of studies on marginal adaptation of root repair materials, several factors such as the design of studies, plane of root sectioning and methods of gap measurement should be considered in order to obtain a better comparison [23].

The second property tested in this study was the solubility. Lack of solubility is a favorable property of the root repair material for obtaining a long term seal without microleakage [24]. Solubility is evaluated by the ISO standards after a period of 24 h, but longer analysis periods may be used [25]. The most widely used time interval is 7 days [9, 25].

In this study, the Biodentine exhibited the highest solubility after 7 days followed by that of the MTA-Angelus then the TheraCal LC. Similar findings were recorded in several previous studies that reported a higher solubility of the Biodentine than MTA [26, 27]. The high solubility of Biodentine could be attributed to the presence of Calcium carbonate and water soluble polycarboxylate in its composition for lowering the water-powder ratio and obtaining good workable cement. The lower solubility of MTA-Angelus than Biodentine could be explained by the absence of Calcium carbonate and presence of setting accelerator in its composition that result in shortening of the setting time [28]. In other studies, the Biodentine exhibited the lowest solubility among the Calcium silicate materials after one day, including the ProRoot MTA [29, 30]. These different results could be related to the differences in the evaluation period as well as conditions of mixing and curing such as water to powder ratio, temperature, environmental humidity and pH, entrapped air and water, the rate of packing and the condensation pressure applied [31].

Regarding the TheraCal LC, it exhibited the lowest solubility compared to the MTA and Biodentine probably due to its immediate setting which is related to the presence of a light-curable resin.

On the other hand, it must bear in mind that the repair materials such as Calcium silicate materials producing Calcium hydroxide or Calcium oxide during setting should present a certain degree of solubility to improve the mineralization process in contact with vital tissue [31].

In the present study, dogs were used as an animal model due to their large, well-developed dental roots that allow better accessibility and visibility. However, in dogs, the furcation lies more superficial within the alveolus (1–2 mm from the cemento-enamel junction) than that of human [32].

Biocompatibility may be evaluated by either cell adhesion assay, cell proliferation, cytotoxicity or inflammatory cell response. In the present study, the inflammatory cell response and radiographic presence or absence of radiolucency adjacent to the perforation site were used to evaluate the biocompatibility after one month and three months. The highest biocompatibility was recorded in the MTA-Angelus subgroup that was followed by that of the Biodentine and TheraCal LC subgroups. In this regard, the MTA-Angelus and Biodentine were less cytotoxic on human dental pulp stem cells (hDPSCs) than TheraCal LC in a previous in vitro study [33]. Moreover, similar findings of the MTA and Biodentine were reported after repairing furcation perforation in dog’s primary teeth [7]. These results could be attributed to the chemical composition of each material. Unlike MTA, the Biodentine has no aluminate phase that does not form ettringite on hydration. Presence of aluminate phase in certain amounts improves the biocompatibility of Portland cement systems [34]. Also, unlike MTA, the Biodentine contains Zirconium oxide that decreases the cell viability [35]. On the other hand, another study reported that addition of Zirconium oxide to the Calcium silicate cement provides a good biocompatibility [36]. Therefore, the exact effect of Zirconium oxide on biocompatibility needs further investigations. In contrast to our findings, Jung et al*. *[37] found that Biodentine is more biocompatible than the MTA on the cells of human periodontal ligament (PDL) after 20 days. This difference might be attributed to the difference of evaluation periods, the type of MTA used and nature of the study (in vitro).

TheraCal LC subgroup exhibited mild to moderate inflammation after one month and moderate to severe inflammation after 3 months of evaluation. These findings mean that TheraCal LC material is less biocompatible than the MTA-Angelus and Biodentine. This may be due to the presence of resin in its composition. These findings are in agreement with other studies [38, 39]. Also the depth of cure should be considered [39].

It is worth to mention that no furcation in any tested subgroup was free of inflammatory cells during both evaluation periods. This could be attributed to the somewhat shorter periods of evaluation. In this regard, various degrees of tissue reactions in contact with the MTA were reported in dogs after 30 days while after 180 days the periodontium was almost free of inflammation [40]. This allows the speculation that a better result might be observed over a longer post-operative period than those of this study.

The radiographic evaluation demonstrates the effectiveness of the tested material on sealing of the perforation and preventing the formation of bony defect adjacent to the perforation site. The results of radiographic evaluation agree with those of the histological findings. The frequency distribution of radiolucency presented in the TheraCal LC subgroup was higher than that of the MTA-Angelus and Biodentine subgroups of both groups with non-significant difference between TheraCal LC and control subgroups. This could be attributed to the lower biocompatibility of the TheraCal LC than that of the MTA-Angelus and Biodentine.

## Conclusion

Unlike Biodentine, TheraCal LC is incapable of alternating the MTA in the furcation perforation repair due to its poor biocompatibility and poor marginal adaptation**.**

## Data Availability

All data used and/or analyzed during this research are available from the corresponding author on reasonable request.

## Abbreviations

MTA: Mineral trioxide aggregate
SEM: Scanning electronic microscope
ISO: International standards organization
PRP: Platelet rich plasma
PRF: Platelet rich fibrin
RMCS: Resin modified calcium silicates
Bis-GMA: Bisphenol A diglycidyl methacrylate
PEGDMA: Polyethylene glycol dimethacrylate
ADA: American Dental Association
ARRIVE: The animal research: Reporting in vivo experiments
EDTA: Ethylenediaminetetraacetic acid
PDL: Periodontal ligament
hDPSCs: Human dental pulp stem cells
